# Personalized Nursing Approaches in Monitoring and Managing Hematoma Risks After Percutaneous Renal Biopsy: A Prospective Observational Study

**DOI:** 10.1155/jonm/9929094

**Published:** 2026-08-17

**Authors:** Di-fei Duan, Min Liu, Deng-yan Ma, Jiao-jiao Zhou, Shen-yi Wei, Lin-jia Yan, Ling Li

**Affiliations:** ^1^ Department of Nephrology, Kidney Research Institute, West China Hospital of Sichuan University, Chengdu, China, wchscu.cn; ^2^ West China School of Nursing, Sichuan University, Chengdu, China, scu.edu.cn; ^3^ Department of Ultrasound, West China Hospital of Sichuan University, Chengdu, China, wchscu.cn; ^4^ Department of Nursing, West China Hospital of Sichuan University, Chengdu, China, wchscu.cn; ^5^ The Nethersole School of Nursing Faculty of Medicine, The Chinese University of Hong Kong, Hong Kong, China, cuhk.edu.hk

**Keywords:** hematoma, nursing, percutaneous renal biopsy, point-of-care ultrasound

## Abstract

**Background:**

This study investigates factors associated with large hematomas after percutaneous renal biopsy (PRB) and describes PRB‐related complications in patients with native kidneys.

**Methods:**

A prospective observational cohort study was conducted from September 2023 to May 2024 on 418 patients undergoing PRB at a nephrology department. The study collected data including demographic details, laboratory data, and preprocedure renal ultrasonography findings. The advanced practice nurse utilized point‐of‐care ultrasound to conduct immediate bedside ultrasound examinations following PRB. Postoperative monitoring involved periodic ultrasound assessments to measure hematoma size at 1, 6, and 24 h after the biopsy, complemented by pain evaluations using the Visual Analog Scale at 6 and 24 h.

**Results:**

The study investigated complications in 418 patients post‐PRB, revealing that 7.7% experienced hematuria and hematoma incidence increased from 45.5% at 1 h to 56.0% at 24 h. ROC curve analysis identified critical cutoffs for predicting significant hematoma growth: 3.05 cm at 1 h (AUC = 0.827) and 4.125 cm at 6 h (AUC = 0.899). Factors significantly linked to larger hematomas at 24 h included activated partial thromboplastin time (APTT) (OR = 1.25, *p* = 0.001), 6‐h hematoma diameter (OR = 2.05, *p* < 0.001), and 24‐h post‐PRB pain score at the puncture site (OR = 1.28, *p* = 0.022).

**Conclusion:**

Higher APTT, larger hematoma diameter at 6 h, and higher puncture‐site pain scores at 24 h were associated with large hematomas (≥ 5 cm) after PRB. These factors may serve as markers to support risk‐stratified postbiopsy monitoring. Further studies are needed to determine whether nursing interventions guided by these markers can reduce complications or improve patient outcomes.

**Implications for Nursing Management:**

Nurse‐led post‐PRB monitoring may incorporate APTT, 6‐h hematoma diameter, and pain assessment to support risk‐informed surveillance. In clinically stable patients without signs of hematoma progression, light ambulation may be considered as an alternative to prolonged strict bed rest.

## 1. Introduction

In recent years, ultrasound technology has significantly enhanced nursing by improving diagnostic precision and patient care [1]. The widespread adoption of point‐of‐care ultrasound (POCUS) allows nurses to perform rapid bedside assessments, enhancing diagnostic accuracy and clinical decision‐making [2]. Enhanced training has strengthened nurses’ ultrasound skills, especially in critical care and emergency departments [2], establishing ultrasound as an essential tool for improving patient outcomes and streamlining clinical workflows [3]. As training programs have matured [4], nurses increasingly use POCUS across specialties such as primary care, perioperative care, and chronic disease management [5], highlighting its growing versatility and importance in modern nursing practice [6–9].

The use of POCUS has expanded in nephrology, supporting the bedside assessment of conditions such as hydronephrosis and kidney stones and the evaluation of hemodialysis access [10]. Its portability and ease of use enhance patient care, while technological advancements and expanded education contribute to improved diagnostic precision [10]. These attributes align with the principles of precision nursing, allowing for more personalized and effective care strategies tailored to individual patient needs. Incorporating precision nursing principles into the management of renal biopsy complications may further enhance patient safety and comfort by promoting individualized care plans that consider patients’ unique clinical profiles and preferences.

Renal biopsy is an invasive procedure that can be categorized into percutaneous, transvenous, laparoscopic, and open surgical approaches. Ultrasound‐guided percutaneous renal biopsy (PRB) has been utilized for over half a century and is now considered the gold standard for diagnosing kidney pathology. It is widely employed for the assessment of renal parenchymal diseases [11]. While PRB is invaluable for diagnosis, it carries inherent risks, including bleeding, pain, infection at the puncture site, and potential damage to adjacent organs. Postpuncture bleeding, particularly along the needle path, is the primary complication associated with PRB [12–14]. The main manifestations of postoperative bleeding include hematuria (microscopic or gross) and perirenal hematoma [15]. In cases where bleeding leads to a decrease in hemoglobin (Hb) levels, some patients may require blood transfusion or interventional hemostasis, resulting in prolonged hospital stays and increased healthcare costs. The reported incidence of bleeding after PRB varies widely across institutions due to differing bleeding criteria [13, 16, 17].

Current risk factors for bleeding after PRB primarily include coagulation disorders, elevated serum creatinine (Scr) levels, and hypertension [18, 19]. Effective management of bleeding complications should prioritize prevention. However, there is no consensus on the necessity of strict bed rest following renal biopsy. Some experts advocate strict bed rest for 6 h after the procedure [20–22], while others recommend close monitoring for 24 h postbiopsy, citing a higher incidence of serious bleeding complications during the 6‐ to 24‐h period [23]. Prolonged strict bed rest may result in emotional distress, back pain, and voiding difficulties, potentially necessitating urinary catheterization, which can adversely affect patient comfort [24, 25].

In this study, we performed bedside ultrasound examinations at 1, 6, and 24 h post‐procedure for patients undergoing PRB of the native kidney. We dynamically assessed changes in the size of perirenal hematomas throughout the bed rest period. In addition, we monitored for the presence of gross hematuria and conducted pain assessments. The primary objective was to identify factors associated with the development of larger hematomas (≥ 5 cm) at 24 h postbiopsy. A secondary objective was to estimate the overall incidence of PRB‐related complications and to evaluate the ability of hematoma diameter at 1 and 6 h to discriminate hematomas ≥ 5 cm at 24 h.

## 2. Materials and Methods

### 2.1. Participants and Settings

A prospective observational cohort study was conducted among patients hospitalized in the Department of Nephrology at our hospital who underwent PRB for native kidneys. The reporting of this observational cohort study followed the STROBE Statement (Supporting File 1). The study included participants who were ① aged ≥ 18 years, ② required PRB based on clinical indications, and ③ provided written informed consent to participate in the study. Exclusion criteria were as follows: ① patients with clinical contraindications to renal biopsy, including uncontrolled hypertension (target blood pressure < 140/90 mmHg before the procedure), coagulation disorders (prothrombin time [PT] exceeding the normal control by > 3 s, APTT prolonged by > 10 s, or platelet count (PLT) < 100 × 10^9^/L), advanced chronic renal changes on prebiopsy ultrasound (e.g., markedly increased cortical echogenicity with loss of corticomedullary differentiation), renal anatomical abnormalities, or severe urinary tract infection; ② patients unable to cooperate with bed rest and postoperative observation; and ③ participation in other interventional clinical trials. The conditions listed under the first exclusion criterion were standard clinical contraindications to PRB rather than study‐specific exclusion criteria [26, 27]. The details of participant recruitment are shown in Supporting Figure 1.

The study included 418 patients who underwent PRB between September 2023 and May 2024. This study was approved by the Ethics Committee of West China Hospital, Sichuan University (Approval No. 2022‐832), and was conducted in accordance with the principles of the Declaration of Helsinki. Written informed consent was obtained from all participants prior to enrollment. In addition to providing written informed consent for the renal biopsy procedure as part of routine clinical care, all participants provided separate written informed consent to participate in this prospective observational study, including postoperative observational monitoring and the collection and use of their clinical and ultrasound data for research purposes.

### 2.2. Data Collection

We collected data on clinical parameters, including sex, age, educational background, and body mass index (BMI). Laboratory data collected included Hb, PLT, Scr, PT, activated partial thromboplastin time (APTT).

Before undergoing PRB, all patients underwent bilateral renal ultrasonography to assess kidney size and cortical thickness. Data were also gathered on the patients’ pathological diagnoses, length of stay, hospitalization costs, and urinary catheterization. Additional variables included the number of needle passes, the number of glomeruli sampled, the experience of the operating physician, whether patients underwent limited mobilization after the initial 6‐h bed‐rest period, CKD stage, and comorbidities.

### 2.3. Renal Biopsy Procedure

Anticoagulant and antiplatelet agents, including aspirin, warfarin, heparin, and direct factor Xa inhibitors, were discontinued 3–7 days prior to the procedure, depending on the pharmacokinetics of each drug [28]. Preoperative patient education was provided by an advanced practice nurse (APN), including instructions on positioning and training in inspiratory apnea during the biopsy procedure. On the day of the procedure, patients continued their antihypertensive medications as scheduled. Systolic blood pressure (SBP) and diastolic blood pressure (DBP) were recorded 30 min before the procedure. Patients were instructed to empty their bladder before the procedure.

All biopsies were performed with the Bard MG1522 Magnum Biopsy Instrument with 16‐gauge biopsy needles (diameter 1.65 mm and length 22 mm) by a well‐trained nephrologist with an ultrasound physician in attendance to assist in real‐time ultrasound guidance. A strict written biopsy protocol was followed.

The puncture site was disinfected and infiltrated with a local anesthetic. Two to three specimens were generally required for light microscopy, electron microscopy, and immunofluorescence [29]. Two to five needle passes were performed to obtain adequate tissue, unless a hematoma developed after an earlier pass.

### 2.4. Postoperative Monitoring and Evaluation

**FIGURE 1:**
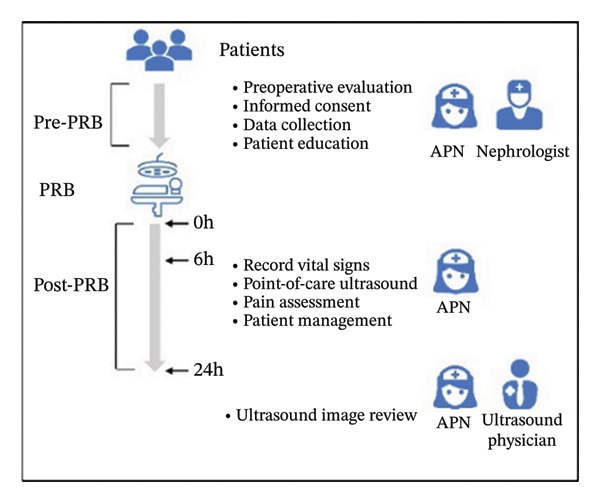
Workflow of the medical team during percutaneous renal biopsy procedures.

All patients remained on strict bed rest for the first 6 h and under in‐hospital observation for 24 h after PRB [30]. Bedside ultrasound examinations were performed at 1, 6, and 24 h to measure perirenal hematoma size. Puncture‐site and lower‐back pain were assessed separately using the Visual Analog Scale (VAS) at 6 and 24 h. After standardized ultrasound training, the APN performed and documented POCUS assessments using a predefined protocol, and all images and measurements were verified by an ultrasound physician. Bleeding complications after PRB, including hematoma, hematuria, requiring blood transfusion, and angiographic intervention were all recorded (details are shown in Figure 1). The primary outcome was defined as a perirenal hematoma with a maximum diameter of ≥ 5 cm at 24 h, based on thresholds used in previous studies [13, 31].

### 2.5. Pain Assessment

Pain assessment was conducted using the VAS, a widely utilized subjective tool for pain measurement. The VAS consists of a 10‐cm horizontal line representing pain intensity, with “0” indicating no pain and “10” indicating the worst possible pain. Following PRB, patients were asked to mark the intensity of their lumbar pain on the VAS. The pain score for each patient was determined by measuring the distance in centimeters from the “no pain” end of the line to the point marked by the patient, thus quantifying the subjective pain experience. In this study, VAS scores were recorded at 6 and 24 h post‐PRB to evaluate patients’ lower back pain as well as puncture site pain [32].

### 2.6. Patient Activity Management

Because there are no specific guidelines on whether 24 h of strict bed rest is necessary, our center adopted a gradual mobilization protocol after PRB. Patients were required to remain supine for the first 6 h, during which the APN used POCUS to assess the perirenal hematoma size. If the hematoma diameter at 6 h was < 5 cm and no concerning clinical signs or symptoms, such as gross hematuria, tachycardia, or hypotension, were present, the APN consulted the supervising physician. Following physician review and approval, patients were permitted light ambulation, including brief bedside activity and voiding in a seated position. They were also permitted to sit upright or turn independently to a lateral position in bed.

### 2.7. Sample Size Calculation

Based on the incidence of perirenal hematoma reported by Bhattacharya et al. [31], the required sample size was estimated using an expected proportion of 41.25%, a two‐sided 95% confidence level, and an absolute precision of 5%. After allowing for 5% incomplete data, the minimum required sample size was 392 participants. All eligible patients were consecutively enrolled during the prespecified recruitment period, resulting in 418 participants with complete data being included in the final analysis.

### 2.8. Statistical Analysis

Normally distributed continuous variables were summarized as means and standard deviations, nonnormally distributed variables as medians (Q1, Q3), and categorical variables as frequencies and percentages. For univariable analyses, we employed the Wilcoxon rank‐sum test, chi‐square test, or Fisher’s exact test depending on variable characteristics. Variables with a *p* value < 0.10 in the univariable analyses were entered into a multivariable logistic regression model to estimate adjusted associations and address potential confounding. Continuous variables entered into the regression model were analyzed on their original scales unless, otherwise, specified. ROC curve analyses were performed separately for the 1‐ and 6‐h hematoma diameters. In an exploratory, data‐driven step, the 6‐h hematoma diameter was selected for inclusion in the multivariable logistic regression model because it showed the larger observed AUC. Participants with incomplete postoperative monitoring data were excluded from the final complete‐case analysis, and no imputation was performed. No data were missing for the variables included in the final analysis. Statistical analyses were performed using R software. Statistical significance was established at a *p* value of less than 0.05.

## 3. Results

### 3.1. Baseline Demographic and Clinical Characteristics

This study included 418 patients with a median age of 42 years and a median BMI of 23.4 kg/m^2^. The cohort consisted of 50.2% females and 49.8% males. More than half of the participants (51.4%) had over 12 years of education, and 77.8% were married. The median SBP was 122 mmHg, and the median DBP was 80 mmHg. Primary glomerular diseases accounted for 57.2% of the cases. Comorbidities were present in 93.5% of the patients, with most cases being in the early stages of CKD (Stages 1–3). The median number of needle passes was 2, and 43.8% of biopsy specimens contained 10–20 glomeruli. Physicians with more than 5 years of PRB experience performed 74.6% of the procedures (Table 1). Among the 418 participants included in the final analysis, no data were missing for the variables analyzed.

**TABLE 1:** Demographical and clinical data of patients undergoing PRB (*n* = 418).

Variables	Total (*n* = 418)	Hematoma diameters ≥ 5 cm at 24 h		*p* Value
		No (*n* = 374)	Yes (*n* = 44)	
Age (median [IQR])	42.00 [31.00, 54.00]	42.00 [31.00, 55.00]	42.00 [28.00, 53.00]	0.380
Sex, *n* (%)				0.028
Female	210 (50.2)	181 (48.4)	29 (65.9)	
Male	208 (49.8)	193 (51.6)	15 (34.1)	
Educational level, *n* (%)				0.869
6 years or below	60 (14.4)	53 (14.2)	7 (15.9)	
7–12 years	143 (34.2)	127 (34.0)	16 (36.4)	
> 12 years	215 (51.4)	194 (51.9)	21 (47.7)	
Marital status, *n* (%)				0.936
Unmarried	93 (22.2)	83 (22.2)	10 (22.7)	
Married	325 (77.8)	291 (77.8)	34 (77.3)	
BMI (median [IQR])	23.43 [20.85, 26.25]	23.51 [20.97, 26.25]	23.04 [20.58, 26.07]	0.341
Systolic blood pressure (median [IQR])	122.00 [112.00, 135.00]	121.00 [111.25, 134.75]	126.50 [115.00, 137.25]	0.189
Diastolic blood pressure (median [IQR])	80.00 [72.25, 87.00]	80.00 [72.00, 87.00]	84.00 [75.00, 91.25]	0.089
Hemoglobin (median [IQR])	130.00 [115.00, 144.75]	130.00 [114.25, 145.00]	125.50 [115.00, 139.75]	0.537
Platelets (median [IQR])	221.00 [173.00, 272.00]	223.00 [173.00, 275.00]	205.00 [175.00, 246.00]	0.293
Serum creatinine (median [IQR])	93.00 [68.25, 136.00]	93.00 [68.00, 136.75]	90.00 [73.75, 122.25]	0.874
Estimated glomerular filtration rate (median [IQR])	77.09[49.16, 103.44]	77.77[49.80, 103.27]	64.40[46.96, 104.54]	0.620
Prothrombin time (median [IQR])	10.80 [10.30, 11.40]	10.70 [10.22, 11.30]	11.10 [10.50, 11.65]	0.017
APTT (median [IQR])	26.85 [25.20, 28.60]	26.80 [25.20, 28.50]	27.95 [26.28, 30.50]	0.008
Pathological types, *n* (%)				0.754
Primary glomerular diseases	239 (57.2)	215 (57.5)	24 (54.5)	
Allergic purpura	15 (3.6)	13 (3.5)	2 (4.5)	
Diabetic nephropathy	25 (6.0)	23 (6.1)	2 (4.5)	
Lupus nephritis	29 (6.9)	25 (6.7)	4 (9.1)	
Minimal change disease	64 (15.3)	59 (15.8)	5 (11.4)	
Others or suboptimal sample quality	46 (11.0)	39 (10.4)	7 (15.9)	
Comorbidities, *n* (%)				0.825
No	27 (6.5)	25 (6.7)	2 (4.5)	
Yes	391 (93.5)	349 (93.3)	42 (95.5)	
CKD stage, *n* (%)				0.447
1	153 (36.6)	139 (37.2)	14 (31.8)	
2	112 (26.8)	99 (26.5)	13 (29.5)	
3	110 (26.3)	99 (26.5)	11 (25.0)	
4	33 (7.9)	27 (7.2)	6 (13.6)	
5	10 (2.4)	10 (2.7)	0 (0.0)	
Renal cortical thickness (median [IQR])	1.40 [1.20, 1.50]	1.40 [1.22, 1.50]	1.35 [1.20, 1.50]	0.636
Operational factors (median [IQR])				
Number of needle passes	2.00 [2.00, 2.00]	2.00 [2.00, 2.00]	2.00 [2.00, 2.00]	0.394
Number of glomeruli, *n* (%)				0.433
0–9 glomeruli	143 (34.2)	127 (34.0)	16 (36.4)	
10–20 glomeruli	183 (43.8)	162 (43.3)	21 (47.7)	
21–30 glomeruli	71 (17.0)	64 (17.1)	7 (15.9)	
> 30 glomeruli	21 (5.0)	21 (5.6)	0 (0.0)	
Operating doctor, *n* (%)				0.928
≤ 5 years	106 (25.4)	96 (23.0)	10 (2.4)	
> 5–10 years	238 (56.9)	211 (50.5)	27 (6.5)	
> 10 years	74 (17.7)	67 (16.0)	7 (1.7)	
Whether engaging in light ambulation, *n* (%)				0.001
No	315 (75.4)	273 (73.0)	42 (95.5)	
Yes	103 (24.6)	101 (27.0)	2 (4.5)	
Hematoma diameter at 1 h (median, [IQR])	0.00 [0.00, 2.80]	0.00 [0.00, 2.49]	5.55 [2.59, 7.66]	< 0.0001
Hematoma diameter at 6 h (median, [IQR])	1.82 [1.82,3.4]	1.50 [0.00, 2.90]	7.11 [4.46, 8.35]	< 0.0001
6‐h post‐PRB pain score at puncture site (median, [IQR])	1 [0,2]	1 [0,2]	1 [0,2]	0.65
24‐h post‐PRB pain score at puncture site (median, [IQR])	0 [0,2]	0 [0,2]	1 [0,3]	0.031
6‐h post‐PRB pain score at low back (median, [IQR])	1 [0,2]	1 [0,2]	1 [0,2]	0.473
24‐h post‐PRB pain score at low back (median, [IQR])	2 [1,3]	2 [1, 3]	2 [1,5]	0.017

### 3.2. Patients With PRB Complications Within 24 h

**FIGURE 2:**
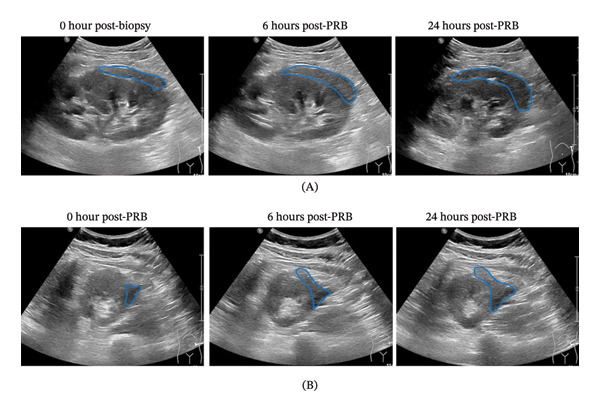
Ultrasound imaging of hematomas postpercutaneous renal biopsy.

**FIGURE 3:**
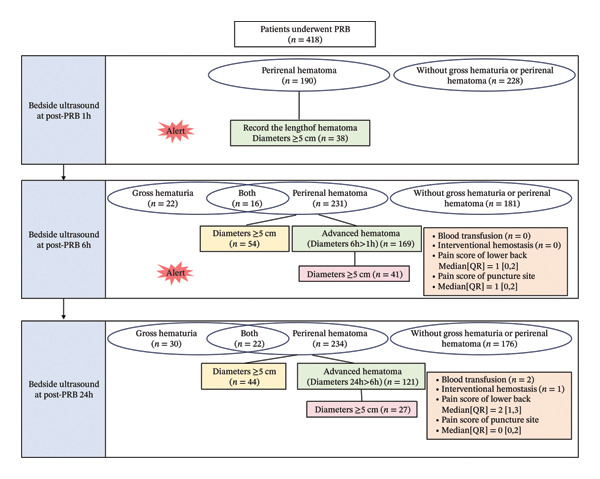
Complications following percutaneous renal biopsy (*n* = 418).

**FIGURE 4:**
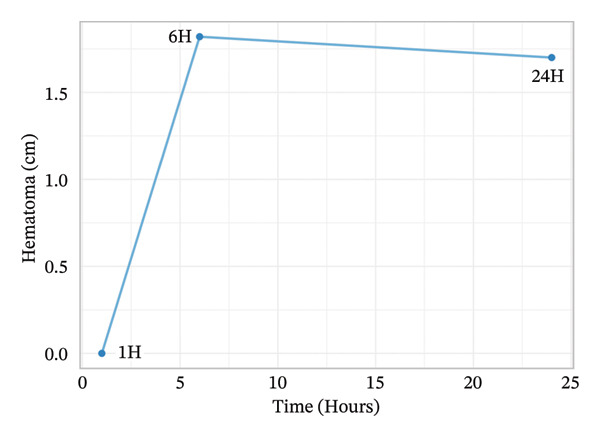
Trends in hematoma size at 1, 6, and 24 h after PRB.

Based on the data presented, hematoma incidence rates at various time points were as follows: 45.5% (*n* = 190) at 1 h, 55.3% (*n* = 231) at 6 h, and 56.0% (*n* = 234) at 24 h. Hematomas measuring 5 cm or greater at 24 h were observed in 10.5% of the cases. Moreover, the hematoma size increased in 28.9% of the patients at 24 h compared to 6 h and 40.4% showed an increase at 6 h compared to 1 h. Figure 2 depicts advanced hematoma in two patients. Ultrasound imaging showed progressively enlarging subcapsular hematomas along the perirenal area. The median low back pain score was 1 (IQR: 0–2) at 6 h and increased to 2 (IQR: 1–3) at 24 h. Two patients (0.5%) required blood transfusion, and one patient (0.2%) underwent angiographic intervention (Figure 3). Additionally, 7.7% of the patients experienced hematuria following PRB. The mean hematoma diameters at 1, 6, and 24 h were 1.70 ± 2.25 cm, 2.23 ± 2.51 cm, and 2.07 ± 2.35 cm, respectively (Figure 4).

### 3.3. ROC Curve Analysis of Hematoma Diameter at 1 and 6 hours for Predicting 24‐Hour Hematoma (≥ 5 cm)

**FIGURE 5:**
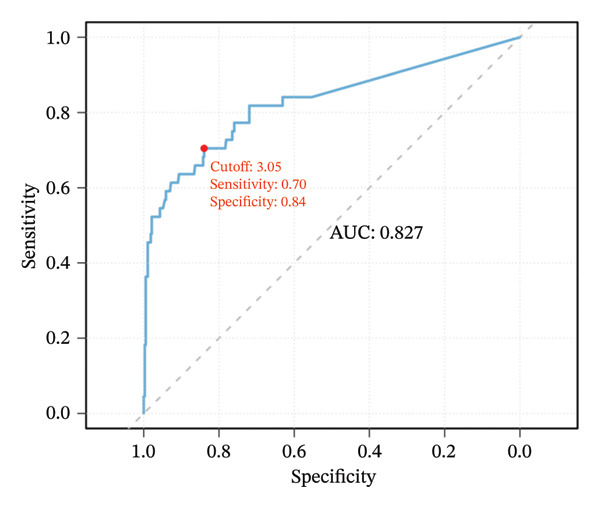
ROC curve for predicting 24‐h hematomas ≥ 5 cm using 1‐h hematoma diameter.

**FIGURE 6:**
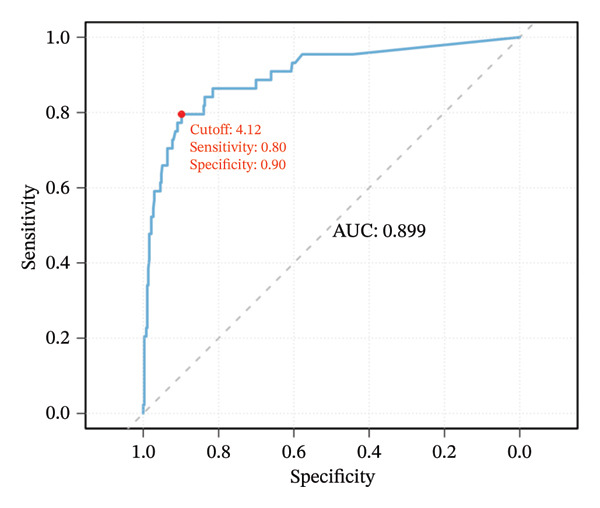
ROC curve for predicting 24‐h hematomas ≥ 5 cm using 6‐h hematoma diameter.

As shown in Figure 5, the area under the receiver operating characteristic curve for the 1‐h hematoma diameter in discriminating hematomas ≥ 5 cm at 24 h was 0.827. The data‐derived cutoff was 3.05 cm, with a sensitivity of 0.70 and a specificity of 0.84. As shown in Figure 6, the corresponding AUC for the 6‐h hematoma diameter was 0.899. The data‐derived cutoff was 4.125 cm, with a sensitivity of 0.898 and a specificity of 0.795. These findings indicate that early hematoma diameter, particularly the 6‐h measurement, showed good discrimination for the presence of a hematoma ≥ 5 cm at 24 h in this cohort. These cutoffs require external validation before clinical application.

### 3.4. Factors Associated With a Hematoma Diameter ≥ 5 cm at 24 hours

The logistic regression analysis identified factors significantly associated with post‐PRB hematoma (≥ 5 cm) at 24 h (Table 2). APTT was associated with higher odds of a large hematoma (OR = 1.25; 95% CI: 1.10–1.42, *p* = 0.001). Larger hematoma diameter at 6 h was also associated with higher odds of this outcome (OR = 2.05; 95% CI: 1.72–2.44, *p* < 0.001). In addition, higher 24‐h puncture‐site pain scores were associated with the presence of a large hematoma (OR = 1.28; 95% CI: 1.04–1.57, *p* = 0.022). Neither the number of needle passes nor baseline renal function demonstrated a statistically significant association with post‐PRB hematoma (≥ 5 cm) at 24 h in the multivariable model (both *p* > 0.05).

**TABLE 2:** Factors associated with hematoma diameters ≥ 5 cm at 24 hours (*n* = 418).

	*p* value	Odds ratio	95% Confidence interval	
			Lower limit	Upper limit
APTT	0.001	1.25	1.10	1.42
Hematoma diameter at 6 h	< 0.001	2.05	1.72	2.44
24‐h post‐PRB pain score at puncture site	0.022	1.28	1.04	1.57

## 4. Discussion

Renal biopsy plays a crucial role in diagnosing and determining the treatment pathway for kidney diseases, though it carries a risk of complications. Specifically, perinephric hematomas are a common complication that warrants close clinical attention. Studies have reported an overall incidence of hematomas ranging from approximately 11%–41.2% [13, 31, 33]. A systematic review has shown that routine imaging after PRB can enhance the detection of hematomas [33]. This is the first study to continuously monitor hematomas at 1, 6, and 24 h post‐PRB by an APN in Chinese patients, revealing hematoma prevalences of 45.5% at 1 h, 55.3% at 6 h, and 56.0% at 24 h. These dynamic incidence rates are consistent with findings by Bhattacharya et al. and Feldmann et al. [31, 34], which further suggest that continuous monitoring of hematomas post‐PRB is essential for improving patient safety. Additionally, the changes in hematoma diameter observed at our monitored time points were more pronounced during the first 6 h after the procedure than during the subsequent 6–24‐h period. This suggests that the first 6 h after the procedure are probably a critical window for monitoring.

The correlation between post‐PRB bleeding risk and hematoma size remains controversial [35]. Nonetheless, many studies have not reported the size of hematomas, complicating the identification of clinically relevant cases. However, in both clinical practice and related research, hematomas with a diameter of 5 cm or larger are typically regarded as critical points of observation [13, 31, 33]. These larger hematomas often indicate a higher clinical risk, potentially leading to significant symptoms or necessitating further intervention.

The results of our multivariate logistic regression analysis underscore significant predictors associated with the formation of larger hematomas (≥ 5 cm) at 24 h post‐PRB. Notably, an increased APTT was found to be significantly correlated with the likelihood of developing larger hematomas. This association suggests that patients with prolonged APTT, indicative of potential coagulation pathway impairments, face a heightened risk of substantial postprocedural bleeding. This is consistent with existing knowledge that coagulation abnormalities contribute to bleeding risks following invasive procedures like PRB [36]. These results further reaffirm the importance of monitoring coagulation indicators such as APTT before PRB. In contrast to some previous reports, our analysis found that the number of needle passes and baseline renal function (estimated glomerular filtration rate (eGFR)/Scr) were not significantly associated with large hematoma formation, whereas multicenter data have linked higher creatinine and more passes with major postbiopsy complications [37]. This discrepancy may reflect procedural and cohort differences: real‐time ultrasound guidance and refined technique can reduce avoidable vascular injury even when additional passes are required [11]. Although advanced kidney dysfunction is classically associated with uremia‐related hemostatic abnormalities and higher bleeding risk, the lack of association with baseline eGFR in our study may be explained by excluding severe chronic renal changes and by preprocedural optimization.

The association between higher postprocedure pain scores at the puncture site and larger hematoma sizes (≥ 5 cm) after PRB has been observed in this study. Patients who report higher pain levels 24 h postprocedure tend to be at greater risk of developing large hematomas. The correlation between pain and active bleeding post‐PRB has also been reported in previous studies by Liu et al. [38]. This is likely due to increased localized trauma during the procedure, resulting in more substantial bleeding and tissue damage, which may manifest as both increased pain and larger hematomas. These findings suggest that patients with elevated post‐PRB pain levels may warrant closer monitoring for potential complications such as large hematomas.

Our study suggests that the 6‐h post‐PRB hematoma diameter may serve as a potential risk marker for a hematoma ≥ 5 cm at 24 h, with an AUC of 0.899 [39]. This assessment may help predict hematomas larger than 5 cm, which are linked to severe clinical outcomes [40]. If 6‐h monitoring is impractical, the 1‐h hematoma measurement serves as a viable alternative, offering reasonable sensitivity and specificity (AUC of 0.827). This flexibility allows for effective patient monitoring and timely intervention in dynamic clinical settings. Moreover, the use of noninvasive POCUS provides objective indicators that can significantly inform clinical decision‐making. By prioritizing the 6‐h measurement for its predictive strength and reserving the 1‐h measurement for situations constrained by logistical challenges, APNs can ensure comprehensive and adaptable patient management. This approach may facilitate risk‐stratified monitoring and support clinical decision‐making in post‐PRB care.

Strict bed rest has traditionally been a cornerstone of post‐PRB patient management [20–23]. In this study, while light ambulation was significant in univariable analysis, it did not demonstrate a significant association with the occurrence of larger hematomas (≥ 5 cm) in the multivariable analysis. This finding aligns with the results from Lucena et al. [41], who observed that reducing patients’ bed rest time from 24 to 8 h did not increase PRB‐related complications. Consequently, our findings suggest that in patients without complications during the first 6 h after PRB, light ambulation may not be associated with an increased risk of bleeding or hematoma enlargement. Permitting such activity may help minimize the discomfort associated with prolonged bed rest and alleviate pain, thereby potentially improving patient comfort. Additionally, allowing light ambulation can reduce the need for catheterization and subsequently lower the risk of catheter‐associated urinary tract infections [41, 42].

The participant screening and exclusion process (Supporting Figure 1) was reviewed to ensure the representativeness and robustness of our findings. Initially, 22 patients were not enrolled because of clinical contraindications, such as uncontrolled hypertension or coagulation disorders, or because they declined to participate. Following enrollment, 23 participants (5.2%) were excluded from the final analysis due to incomplete monitoring. These exclusions were primarily due to random logistical factors (e.g., concurrent clinical tests or temporary unavailability) and patient withdrawal or refusal to continue the protocol, rather than postoperative complications, thereby minimizing selection bias. The final sample of 418 exceeded the prespecified minimum required sample size of 392. Therefore, the exclusions did not reduce the analyzable sample below the prespecified minimum requirement.

Several limitations of this study should be acknowledged. First, as a single‐center study conducted within a specific patient population and institutional practice framework (e.g., eligibility/contraindication criteria for PRB and postbiopsy monitoring pathways), the generalizability of our findings to other clinical settings may be limited. Second, while approximately 90% of the hemorrhagic complications are reported to occur within the first 24 h post‐PRB [43], our study did not perform ultrasound examinations beyond this window; thus, delayed bleeding complications may have gone undetected. Third, although baseline blood pressure was strictly controlled, the absence of continuous 24‐h monitoring precludes a definitive assessment of blood pressure variability as an alternative explanation for hematoma growth. Similarly, despite the discontinuation of high‐risk anticoagulants, the potential confounding effects of other complex comedications were not fully adjusted for in the regression model due to the high prevalence of comorbidities in our cohort. Finally, our study primarily focused on ultrasound‐assessed hematoma size and did not include a longitudinal investigation of postbiopsy hematuria trends. Future multicenter studies with larger, more diverse cohorts and extended follow‐up are warranted to validate and expand upon these findings.

### 4.1. Implications for Nursing Management

These findings suggest a potential role for APN‐performed POCUS in risk‐informed monitoring after PRB. In this cohort, hematoma diameter at 6 h was associated with the presence of a larger hematoma (≥ 5 cm) at 24 h and may help identify patients who require closer observation. Consistent with KHA‐CARI guidance, which does not suggest routine postbiopsy ultrasound for asymptomatic hematomas in uncomplicated or low‐risk patients, a selective, risk‐stratified imaging approach may be more appropriate than universal screening [44]. Among clinically stable patients without early complications, light ambulation was not associated with hematoma enlargement; however, its effects on urinary retention, discomfort, and recovery were not evaluated in this study. Evidence from other clinical settings suggests that POCUS may offer economic and patient‐experience benefits [45, 46], but these outcomes require evaluation specifically in post‐PRB care. Before broader implementation, nurse‐led POCUS pathways should be prospectively assessed and supported by institution‐specific credentialing and competency assessment for APNs, standardized image acquisition, hematoma measurement and documentation, predefined escalation criteria, secure image archiving, and periodic expert review and quality audit [47]. Staffing and workflow arrangements should also clarify responsibility for scheduled assessments, clinical handover and after‐hours coverage, and access to nephrology or ultrasound specialists when findings are uncertain or concerning.

## 5. Conclusion

This study found that APTT and 6‐h hematoma diameter were independently associated with the development of a larger hematoma (≥ 5 cm) within 24 h after PRB. Early identification of patients at increased risk based on these factors may support timely monitoring and clinical decision‐making. Among clinically stable patients without early postprocedural complications, light ambulation after the initial 6‐h bed‐rest period was not associated with hematoma enlargement. These findings may inform risk‐stratified postbiopsy monitoring although further multicenter studies are needed to confirm their clinical applicability.

## Author Contributions

Di‐fei Duan and Min Liu: conceptualization, data curation, formal analysis, investigation, methodology, visualization, and writing–original draft preparation. Deng‐yan Ma and Jiao‐jiao Zhou: investigation, project administration, resources, and validation. Lin‐jia Yan and Shen‐yi Wei: resources and supervision. Ling Li: conceptualization, investigation, methodology, project administration, validation, supervision, and writing–review and editing.

## Funding

The authors received no financial support for the research, authorship, and/or publication of this article.

## Conflicts of Interest

The authors declare no conflicts of interest.

## Supporting Information

Additional supporting information can be found online in the Supporting Information section.

## Supporting information


**Supporting Information 1** Supporting Figure 1. Participant recruitment flow diagram showing the numbers of participants approached, excluded before enrollment, providing informed consent, excluded from the final analysis, and included in the final analysis.

**Supporting Information 2** Supporting File 1. Completed STROBE Statement checklist for this observational study, with the corresponding manuscript page and line numbers provided for each reporting item.

## Data Availability

The data that support the findings of this study are available from the corresponding author upon reasonable request.

## References

[1] Kearns M. , Brennan P. , and Buckley T. , Nurse Practitioners’ Use of Diagnostic Imaging: A Scoping Review, Journal of Clinical Nursing. (2024) 33, no. 2, 432–453, 10.1111/jocn.16874. 37953490

[2] Brotfain E. , Erblat A. , Luft P. et al., Nurse-Performed Ultrasound Assessment of Gastric Residual Volume and Enteral Nasogastric Tube Placement in the General Intensive Care Unit, Intensive and Critical Care Nursing. (2022) 69, 10.1016/j.iccn.2021.103183. 34924254

[3] Galen B. , Baron S. , Young S. , Hall A. , Berger-Spivack L. , and Southern W. , Reducing Peripherally Inserted Central Catheters and Midline Catheters by Training Nurses in Ultrasound-Guided Peripheral Intravenous Catheter Placement, BMJ Quality and Safety. (2020) 29, no. 3, 245–249, 10.1136/bmjqs-2019-009923. 31582569

[4] Ducker G. and Mukhtyar C. B. , Specialist Nurse Training in Ultrasonography for the Diagnosis of Giant Cell Arteritis, Rheumatology. (2023) 62, no. 5, e142–e143, 10.1093/rheumatology/keac591. 36218418

[5] Yamada T. , Ehara J. , Funakoshi H. , Endo K. , and Kitano Y. , Effectiveness of Point of Care Ultrasound (POCUS) Simulation Course and Skills Retention for Japanese Nurse Practitioners, BMC Nursing. (2023) 22, no. 1, 10.1186/s12912-023-01183-2. PMC9872333 36691022

[6] Wiley B. M. , Borlaug B. A. , and Kane G. C. , Lung Ultrasound in Heart Failure: Envisioning Handheld Ultrasound to Empower Nurses and Transform Health Care∗, Journal of the American College of Cardiology. (2022) 80, no. 5, 524–526. 35902176 10.1016/j.jacc.2022.05.021

[7] Sugiyama S. , Asakura K. , and Takada N. , Japanese Nurse Practitioners′ Legal Liability Ambiguity Regarding Their Medical Practice: A Qualitative Study, BMC Nursing. (2020) 19, no. 1, 10.1186/s12912-020-00458-2. PMC7346369 32669968

[8] Sun J. , Li Q. , Wu X. , Wang X. , and Liu D. , Nurse-Performed Ultrasound: A New Weapon Against COVID-19, Critical Care. (2020) 24, no. 1, 10.1186/s13054-020-03160-6. PMC7358560 32665002

[9] Henry-Tillman R. , Kabongo M. , Laryea J. et al., The Ability to Look: Management of Breast Disease in the Democratic Republic of the Congo Using Smart Ultrasound Technology, Journal of the American College of Surgeons. (2021) 232, no. 4, 636–640, 10.1016/j.jamcollsurg.2020.12.008. 33348015

[10] Ross D. W. , Moses A. A. , and Niyyar V. D. , Point-Of-Care Ultrasonography in Nephrology Comes of Age, Clinical Kidney Journal. (2022) 15, no. 12, 2220–2227, 10.1093/ckj/sfac160. 36381376 PMC9664573

[11] Granata A. , Distefano G. , Pesce F. et al., Performing an Ultrasound-Guided Percutaneous Needle Kidney Biopsy: An up-to-Date Procedural Review, Diagnostics. (2021) 11, no. 12, 10.3390/diagnostics11122186. PMC8700183 34943422

[12] Lees J. S. , McQuarrie E. P. , Mordi N. , Geddes C. C. , Fox J. G. , and Mackinnon B. , Risk Factors for Bleeding Complications After nephrologist-performed Native Renal Biopsy, Clinical Kidney Journal. (2017) 10, no. 4, 573–577, 10.1093/ckj/sfx012. 28852497 PMC5570080

[13] Li F. F. , Guan Y. , Li T. et al., Analysis of Hemorrhage Upon Ultrasound-Guided Percutaneous Renal Biopsy in China: A Retrospective Study, International Urology and Nephrology. (2024) 56, no. 5, 1713–1720, 10.1007/s11255-023-03860-2. 37991602 PMC11001650

[14] Ferguson C. , Winters S. , Jackson S. , McToal M. , and Low G. , A Retrospective Analysis of Complication and Adequacy Rates of Ultrasound-Guided Native and Transplant Non-Focal Renal Biopsies, Abdominal Radiology. (2018) 43, no. 8, 2183–2189, 10.1007/s00261-017-1405-z. 29159524

[15] Palsson R. , Short S. A. , Kibbelaar Z. A. et al., Bleeding Complications After Percutaneous Native Kidney Biopsy: Results From the Boston Kidney Biopsy Cohort, Kidney International Reports. (2020) 5, no. 4, 511–518, 10.1016/j.ekir.2020.01.012. 32274455 PMC7136322

[16] Oner S. , Okumus M. M. , Demirbas M. et al., Factors Influencing Complications of Percutaneous Nephrolithotomy: A Single-Center Study, Urology Journal. (2015) 12, no. 5, 2317–2323. 26571313

[17] Korbet S. M. , Volpini K. C. , and Whittier W. L. , Percutaneous Renal Biopsy of Native Kidneys: A Single-Center Experience of 1,055 Biopsies, American Journal of Nephrology. (2014) 39, no. 2, 153–162, 10.1159/000358334. 24526094

[18] Anpalahan A. , Malacova E. , Hegerty K. et al., Bleeding Complications of Percutaneous Kidney Biopsy: Does Gender Matter?, Kidney360. (2021) 2, no. 8, 1308–1312, 10.34067/kid.0002432021. 35369661 PMC8676397

[19] Manno C. , Strippoli G. F. , Arnesano L. et al., Predictors of Bleeding Complications in Percutaneous Ultrasound-Guided Renal Biopsy, Kidney International. (2004) 66, no. 4, 1570–1577, 10.1111/j.1523-1755.2004.00922.x. 15458453

[20] Carrington C. P. , Williams A. , Griffiths D. F. , Riley S. G. , and Donovan K. L. , Adult Day-Case Renal Biopsy: A Single-Centre Experience, Nephrology Dialysis Transplantation. (2011) 26, no. 5, 1559–1563, 10.1093/ndt/gfq571. 20858764

[21] Pakfetrat M. , Malekmakan L. , Hamidianjahromi A. et al., Frequency and Timing of Renal Biopsy Complications, Caspian J Intern Med. (2024) 15, no. 1, 96–100. 38463932 10.22088/cjim.15.1.10 PMC10921104

[22] Schorr M. , Roshanov P. S. , Weir M. A. , and House A. A. , Frequency, Timing, and Prediction of Major Bleeding Complications From Percutaneous Renal Biopsy, Can J Kidney Health Dis. (2020) 7, 10.1177/2054358120923527. PMC7251654 32547772

[23] Schnuelle P. , Renal Biopsy for Diagnosis in Kidney Disease: Indication, Technique, and Safety, Journal of Clinical Medicine. (2023) 12, no. 19, 10.3390/jcm12196424. PMC10573674 37835066

[24] Giordano F. , Mitrotti A. , Losurdo A. et al., Effect of Music Therapy Intervention on Anxiety and Pain During Percutaneous Renal Biopsy: A Randomized Controlled Trial, Clinical Kidney Journal. (2023) 16, no. 12, 2721–2727, 10.1093/ckj/sfad246. 38046004 PMC10689136

[25] Oliveira M. C. , Flores F. D. S. , Barbosa F. M. , Fujii C. D. C. , Rabelo-Silva E. R. , and Lucena AdF. , Evaluation of Percutaneous Renal Biopsy Complications Based on Outcomes and Indicators of the Nursing Outcomes Classification, Rev Lat Am Enfermagem. (2021) 29, 10.1590/1518-8345.3759.3415. PMC8253370 34231785

[26] Madaio M. P. , Renal Biopsy, Kidney International. (1990) 38, no. 3, 529–543, 10.1038/ki.1990.236. 2232496

[27] Najafian B. , Lusco M. A. , Alpers C. E. , and Fogo A. B. , Approach to Kidney Biopsy: Core Curriculum 2022, American Journal of Kidney Diseases. (2022) 80, no. 1, 119–131, 10.1053/j.ajkd.2021.08.024. 35125261

[28] Luciano R. L. and Moeckel G. W. , Update on the Native Kidney Biopsy: Core Curriculum 2019, American Journal of Kidney Diseases. (2019) 73, no. 3, 404–415, 10.1053/j.ajkd.2018.10.011. 30661724

[29] Chang A. , Gibson I. W. , Cohen A. H. , Weening J. J. , Jennette J. C. , and Fogo A. B. , A Position Paper on Standardizing the Nonneoplastic Kidney Biopsy Report, Clinical Journal of the American Society of Nephrology. (2012) 7, no. 8, 1365–1368, 10.2215/cjn.02300312. 22798541

[30] Ultrasound Medicine Branch of the Shanghai Association of Non-Public Medical Institutions , Practice Guidelines for Percutaneous Ultrsound-Guided Renal Biopsy, Chinese Journal of Medical Ultrasound. (2021) 18, no. 11, 1023–1043, (in Chinese).

[31] Bhattacharya S. , Nagaraju S. P. , Prabhu R. A. et al., Sonological Predictors of Complications of Percutaneous Renal Biopsy-a Prospective Observational Study, Irish Journal of Medical Science. (2024) . 10.1007/s11845-024-03753-y PMC11450080 38995486

[32] Bijur P. E. , Silver W. , and Gallagher E. J. , Reliability of the Visual Analog Scale for Measurement of Acute Pain, Academic Emergency Medicine. (2001) 8, no. 12, 1153–1157, 10.1111/j.1553-2712.2001.tb01132.x. 11733293

[33] Poggio E. D. , McClelland R. L. , Blank K. N. et al., Systematic Review and Meta-Analysis of Native Kidney Biopsy Complications, Clinical Journal of the American Society of Nephrology. (2020) 15, no. 11, 1595–1602, 10.2215/cjn.04710420. 33060160 PMC7646247

[34] Feldmann Y. , Böer K. , Wolf G. , and Busch M. , Complications and Monitoring of Percutaneous Renal Biopsy- a Retrospective Study, Clinical Nephrology. (2018) 89, no. 4, 260–268, 10.5414/cn109223. 29154749

[35] Lubas A. , Wojtecka A. , Smoszna J. , Koziński P. , Frankowska E. , and Niemczyk S. , Hemodynamic Characteristics and the Occurrence of Renal biopsy-related Arteriovenous Fistulas in Native Kidneys, International Urology and Nephrology. (2016) 48, no. 10, 1667–1673, 10.1007/s11255-016-1411-z. 27580732 PMC5031753

[36] Pombas B. , Rodríguez E. , Sánchez J. et al., Risk Factors Associated with Major Complications After Ultrasound-Guided Percutaneous Renal Biopsy of Native Kidneys, Kidney and Blood Pressure Research. (2020) 45, no. 1, 122–130, 10.1159/000504544. 31822004

[37] Andrulli S. , Rossini M. , Gigliotti G. et al., The Risks Associated With Percutaneous Native Kidney Biopsies: A Prospective Study, Nephrology Dialysis Transplantation. (2022) 38, no. 3, 655–663, 10.1093/ndt/gfac177. PMC9976765 35587882

[38] Liu W. , Jin C. , Lian Q. et al., Ultrasound-Guided Lauromacrogol Injection for the Treatment of Active Bleeding After Renal Biopsy, Frontiers in Pharmacology. (2021) 12, 10.3389/fphar.2021.723634. PMC8733379 35002689

[39] Hosmer Jr, D. W. , Lemeshow S. , and Sturdivant R. X. , Applied Logistic Regression, 2013, John Wiley & Sons.

[40] Xu D. M. , Chen M. , Zhou F. , and Zhao M. , Risk Factors for Severe Bleeding Complications in Percutaneous Renal Biopsy, The American Journal of the Medical Sciences. (2017) 353, no. 3, 230–235, 10.1016/j.amjms.2016.12.019. 28262208

[41] de Fátima Lucena A. , Oliveira M. C. , and Manfro R. C. , Reduction of Patients′ Bed Rest Time After Percutaneous Renal Biopsy Evaluated by the Nursing Outcomes Classification: Randomized Clinical Trial, Int J Nurs Knowl. (2024) 35, no. 3, 308–316. 37676727 10.1111/2047-3095.12447

[42] Ishikawa E. , Nomura S. , Obe T. et al., How Long is Strict Bed Rest Necessary After Renal Biopsy?, Clinical and Experimental Nephrology. (2009) 13, no. 6, 594–597, 10.1007/s10157-009-0206-2. 19578930

[43] Ubara Y. , Kawaguchi T. , Nagasawa T. et al., Kidney Biopsy Guidebook 2020 in Japan, Clinical and Experimental Nephrology. (2021) 25, no. 4, 325–364, 10.1007/s10157-020-01986-6. 33606126 PMC7966701

[44] MacGinley R. , Champion De Crespigny P. J. , Gutman T. et al., KHA-CARI Guideline Recommendations for Renal Biopsy, Nephrology. (2019) 24, no. 12, 1205–1213, 10.1111/nep.13662. 31490584

[45] Lentz B. , Fong T. , Rhyne R. , and Risko N. , A Systematic Review of the Cost-Effectiveness of Ultrasound in Emergency Care Settings, Ultrasound J. (2021) 13, no. 1, 10.1186/s13089-021-00216-8. PMC7943664 33687607

[46] Balmuth E. A. , Luan D. , Jannat-Khah D. , Evans A. , Wong T. , and Scales D. A. , Point-Of-Care Ultrasound (POCUS): Assessing Patient Satisfaction and Socioemotional Benefits in the Hospital Setting, PLoS One. (2024) 19, no. 2, 10.1371/journal.pone.0298665. PMC10871481 38363766

[47] Koratala A. , Argaiz E. R. , Romero-González G. et al., Point-Of-Care Ultrasound Training in Nephrology: A Position Statement by the International Alliance for POCUS in Nephrology, Clinical Kidney Journal. (2024) 17, no. 11, 10.1093/ckj/sfae245. PMC11536759 39502372

