# Supplementary material for: Personalized Nursing Approaches in Monitoring and Managing Hematoma Risks After Percutaneous Renal Biopsy: A Prospective Observational Study
Source: J Nurs Manag. 2026 Aug 17;2026:9929094. doi: 10.1155/jonm/9929094 (PMC13479113; doi:10.1155/jonm/9929094)
Supplement: Supplementary file 1 [file JONM-2026-9929094-s001.docx]

Supplementary Figure 1: Participant Recruitment Flow Diagram for the Study.

Total subjects approached: n=463

Excluded before enrollment:

a. Did not meet inclusion criteria (n=10)

b: Refused participation (n=12)

Informed consent obtained: n=441

Excluded from final analysis: (n=23)

Hematomas monitoring was not conducted at the designated times because patients were either unavailable, refused, or were moved for other tests.

Included in final analysis: n=418
