# Supplementary material for: Personalized Nursing Approaches in Monitoring and Managing Hematoma Risks After Percutaneous Renal Biopsy: A Prospective Observational Study
Source: J Nurs Manag. 2026 Aug 17;2026:9929094. doi: 10.1155/jonm/9929094 (PMC13479113; doi:10.1155/jonm/9929094)
Supplement: Supplementary file 2 [file JONM-2026-9929094-s002.docx]

STROBE Statement—checklist of items that should be included in reports of observational studies

|  | Item No. | Recommendation | Page  No. | Relevant text from manuscript |
| --- | --- | --- | --- | --- |
| **Title and abstract** | 1 | (*a*) Indicate the study’s design with a commonly used term in the title or the abstract | 1,2 | Lines 1, 31-60 |
|  |  | (*b*) Provide in the abstract an informative and balanced summary of what was done and what was found | 2 | Lines 31-59 |
| Introduction | | | |  |
| Background/rationale | 2 | Explain the scientific background and rationale for the investigation being reported | 4-5 | Lines 63-105 |
| Objectives | 3 | State specific objectives, including any prespecified hypotheses | 5 | Lines 110-114 |
| Methods | | | |  |
| Study design | 4 | Present key elements of study design early in the paper | 6 | Lines 118-120 |
| Setting | 5 | Describe the setting, locations, and relevant dates, including periods of recruitment, exposure, follow-up, and data collection | 6-8 | Lines 117-184 |
| Participants | 6 | (*a*) *Cohort study*—Give the eligibility criteria, and the sources and methods of selection of participants. Describe methods of follow-up  *Case-control study*—Give the eligibility criteria, and the sources and methods of case ascertainment and control selection. Give the rationale for the choice of cases and controls  *Cross-sectional study*—Give the eligibility criteria, and the sources and methods of selection of participants | 6,9,10 | Lines 121-143, 177–187, 201–210; Supplementary Figure 1 |
|  |  | (*b*) *Cohort study*—For matched studies, give matching criteria and number of exposed and unexposed  *Case-control study*—For matched studies, give matching criteria and the number of controls per case | NA | NA |
| Variables | 7 | Clearly define all outcomes, exposures, predictors, potential confounders, and effect modifiers. Give diagnostic criteria, if applicable | 5,7-9 | Lines 110–115, 145–155, 177–210 |
| Data sources/ measurement | 8* | For each variable of interest, give sources of data and details of methods of assessment (measurement). Describe comparability of assessment methods if there is more than one group | *7-9* | Lines 143–153, 156–198, 201–210 |
| Bias | 9 | Describe any efforts to address potential sources of bias | 8-10 | Lines 181–183, 216-218, 224-226, and 231-233 |
| Study size | 10 | Explain how the study size was arrived at | 9 | Lines 212-217 |

Continued on next page

| Quantitative variables | 11 | Explain how quantitative variables were handled in the analyses. If applicable, describe which groupings were chosen and why | 9 | Lines 220-227 |
| --- | --- | --- | --- | --- |
| Statistical methods | 12 | (*a*) Describe all statistical methods, including those used to control for confounding | 9-11 | Lines 220-235 |
|  |  | (*b*) Describe any methods used to examine subgroups and interactions | NA | NA |
|  |  | (*c*) Explain how missing data were addressed | 10 | Linse 231-233 |
|  |  | (*d*) *Cohort study*—If applicable, explain how loss to follow-up was addressed  *Case-control study*—If applicable, explain how matching of cases and controls was addressed  *Cross-sectional study*—If applicable, describe analytical methods taking account of sampling strategy | 10,15 | Lines 231-233, and 370–377; Supplementary Figure 1 |
|  |  | (*e*) Describe any sensitivity analyses | NA | NA |
| Results | | | | |
| Participants | 13* | (a) Report numbers of individuals at each stage of study—eg numbers potentially eligible, examined for eligibility, confirmed eligible, included in the study, completing follow-up, and analysed | 6 | Line 134 (Supplementary Figure 1) |
|  |  | (b) Give reasons for non-participation at each stage | 6,15 | Lines 370–377; Line 134 (Supplementary Figure 1) |
|  |  | (c) Consider use of a flow diagram | 6 | Line 134 (Supplementary Figure 1) |
| Descriptive data | 14* | (a) Give characteristics of study participants (eg demographic, clinical, social) and information on exposures and potential confounders | 10 | Lines 238-248 |
|  |  | (b) Indicate number of participants with missing data for each variable of interest | 10 | Lines 231-233;247-248 |
|  |  | (c) *Cohort study*—Summarise follow-up time (eg, average and total amount) | 8 | Lines 177-180 |
| Outcome data | 15* | *Cohort study*—Report numbers of outcome events or summary measures over time | *11* | Lines 250-263 |
|  |  | *Case-control study—*Report numbers in each exposure category, or summary measures of exposure | NA | NA |
|  |  | *Cross-sectional study—*Report numbers of outcome events or summary measures | NA | NA |
| Main results | 16 | (*a*) Give unadjusted estimates and, if applicable, confounder-adjusted estimates and their precision (eg, 95% confidence interval). Make clear which confounders were adjusted for and why they were included | 10,11 | Lines 238–285; |
|  |  | (*b*) Report category boundaries when continuous variables were categorized | 8,11 | Lines 185-187;267-277 |
|  |  | (*c*) If relevant, consider translating estimates of relative risk into absolute risk for a meaningful time period | NA | NA |

Continued on next page

| Other analyses | 17 | Report other analyses done—eg analyses of subgroups and interactions, and sensitivity analyses | 11 | Lines 267–274 |
| --- | --- | --- | --- | --- |
| Discussion | | | | |
| Key results | 18 | Summarise key results with reference to study objectives | 12 | Line 287-301 |
| Limitations | 19 | Discuss limitations of the study, taking into account sources of potential bias or imprecision. Discuss both direction and magnitude of any potential bias | 16 | Line 381-397 |
| Interpretation | 20 | Give a cautious overall interpretation of results considering objectives, limitations, multiplicity of analyses, results from similar studies, and other relevant evidence | 12-15 | Line 303-418 |
| Generalisability | 21 | Discuss the generalisability (external validity) of the study results | 15 | Lines 381–385 |
| Other information | |  | | |
| Funding | 22 | Give the source of funding and the role of the funders for the present study and, if applicable, for the original study on which the present article is based | 17 | No funding |

*Give information separately for cases and controls in case-control studies and, if applicable, for exposed and unexposed groups in cohort and cross-sectional studies.

**Note:** An Explanation and Elaboration article discusses each checklist item and gives methodological background and published examples of transparent reporting. The STROBE checklist is best used in conjunction with this article (freely available on the Web sites of PLoS Medicine at http://www.plosmedicine.org/, Annals of Internal Medicine at http://www.annals.org/, and Epidemiology at http://www.epidem.com/). Information on the STROBE Initiative is available at www.strobe-statement.org.
